# Supplementary material for: The Association Between Cadmium Exposure and Prostate Cancer: An Updated Systematic Review and Meta-Analysis
Source: Int J Environ Res Public Health. 2024 Nov 19;21(11):1532. doi: 10.3390/ijerph21111532 (PMC11593822; doi:10.3390/ijerph21111532)
Supplement: Supplementary file 1 [file ijerph-21-01532-s001.zip › ijerph-3217653-supplementary.pdf]

Supplementary Materials

Table S1. Search strategy *.

Database	Search Details
PubMed	(cadmium[MeSH Terms]) AND (prostate cancer[MeSH Terms])
Web of Science	cadmium (Topic) and prostate cancer (Topic)
Scopus	(cadmium) AND (prostate AND cancer)

* The results obtained from this research are based on the date 1.06.2015 until 28.05.2024.

PECO statement

Participants/population

Inclusion: Human male population from hospital and community.

Exclusion: People combined with other prostate diseases.

Exposure(s)

Cadmium exposure (Residential; Occupational; Dietary)

Comparator(s)/control

Subjects with higher than lower/no exposure of cadmium than exposure group

Main outcome(s)

Prostate cancer

Table S2. New Castle Ottawa Scale of the studied selected of the Cohort studies.

Cohort Study	Representativeness of the Exposed Cohort	Selection of the Unexposed Cohort	Ascertainment of Exposure	Outcome of Interest Not Present at Start of Study	Control for		Assessment of Outcome	Follow-Up Long Enough for Outcomes to Occur	Adequacy of Follow Up of Cohorts	Total Quality Score
					Important Factor	Additional Factors				
Nyqvist F, 2017 Sweden	1	1	1	0	0	0	1	1	1	6
Lequy E, 2023 France	1	0	1	1	1	1	1	1	0	7
Eriksen KT, 2015 Denmark	1	1	1	1	1	1	1	1	1	9

Table S3. New Castle Ottawa Scale of the studied selected of the Case-Control studies.

Case-Control Study	Adequate Definition of Cases	Representativeness of Cases	Selection of Controls	Definition of Controls	Comparability		Ascertainment Exposure	Same Method of Ascertainment for Cases and Controls	Non-Response Rate	Total Quality Score
					Important Factor	Additional Factors				
Bede-Ojimadu O, 2023 Nigeria	1	0	0	1	1	1	1	1	1	7
Li J, 2009 USA	1	1	1	1	1	1	1	1	0	8
Aronson KJ, 1995 Canada	1	1	1	1	1	1	1	1	1	9
Elghany NA, 1990 USA	1	1	1	0	1	0	1	1	1	7

Table S4. New Castle Ottawa Scale of the studied selected of the Cross-sectional studies.

Cross Sectional Study	Representativeness of the Sample	Sample Size	Non-Respondents	Ascertainment of the Exposure	Control for Potential Confounders	Assessment of Outcome	Statistical Test	Total Quality Score
Cao H, 2023 USA	1	1	0	1	1	1	1	6

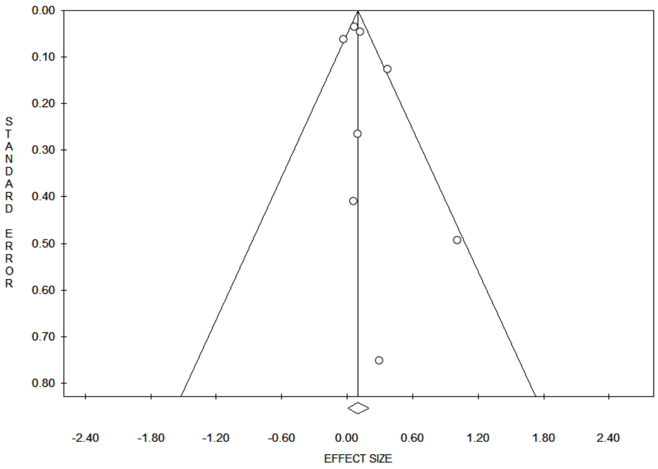

(**a**)

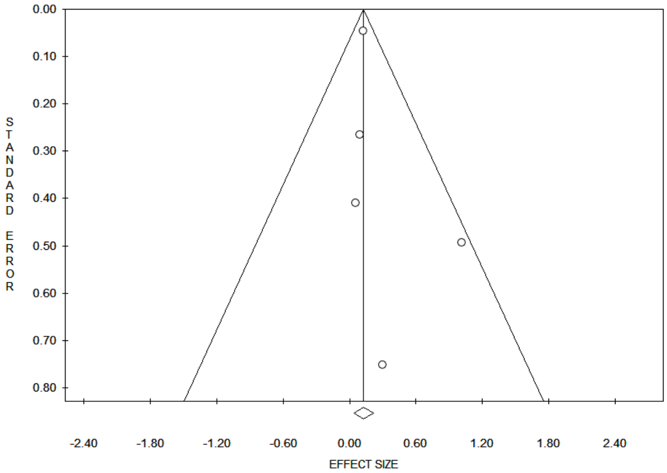

(**b**)

Figure S1. Funnel plot of publication bias of the association between Cd exposure and PCa of Europe articles. (**a**) from previous meta-analysis and new, (**b**) from previous meta-analysis.
